# Remotely sensing harmful algal blooms in the Red Sea

**DOI:** 10.1371/journal.pone.0215463

**Published:** 2019-04-16

**Authors:** Elamurugu Alias Gokul, Dionysios E. Raitsos, John A. Gittings, Abdulsalam Alkawri, Ibrahim Hoteit

**Affiliations:** 1 Physical Science and Engineering Division, King Abdullah University of Science and Technology (KAUST), Thuwal, Kingdom of Saudi Arabia; 2 Remote Sensing Group, Plymouth Marine Laboratory (PML), Plymouth, Devon, United Kingdom; 3 Department of Biology, National and Kapodistrian University of Athens (NKUA), Athens, Greece; 4 Department of Marine Biology, Hodeidah University, Al Hodeidah, Yemen; University of Siena, ITALY

## Abstract

Harmful Algal Blooms (HABs) are of global concern, as their presence is often associated with socio-economic and environmental issues including impacts on public health, aquaculture and fisheries. Therefore, monitoring the occurrence and succession of HABs is fundamental for managing coastal regions around the world. Yet, due to the lack of adequate *in situ* measurements, the detection of HABs in coastal marine ecosystems remains challenging. Sensors on-board satellite platforms have sampled the Earth synoptically for decades, offering an alternative, cost-effective approach to routinely detect and monitor phytoplankton. The Red Sea, a large marine ecosystem characterised by extensive coral reefs, high levels of biodiversity and endemism, and a growing aquaculture industry, is one such region where knowledge of HABs is limited. Here, using high-resolution satellite remote sensing observations (1km, MODIS-Aqua) and a second-order derivative approach, in conjunction with available *in situ* datasets, we investigate for the first time the capability of a remote sensing model to detect and monitor HABs in the Red Sea. The model is able to successfully detect and generate maps of HABs associated with different phytoplankton functional types, matching concurrent *in situ* data remarkably well. We also acknowledge the limitations of using a remote-sensing based approach and show that regardless of a HAB’s spatial coverage, the model is only capable of detecting the presence of a HAB when the *Chl-a* concentrations exceed a minimum value of ~ 1 mg m^-3^. Despite the difficulties in detecting HABs at lower concentrations, and identifying species toxicity levels (only possible through *in situ* measurements), the proposed method has the potential to map the reported spatial distribution of several HAB species over the last two decades. Such information is essential for the regional economy (i.e., aquaculture, fisheries & tourism), and will support the management and sustainability of the Red Sea’s coastal economic zone.

## Introduction

Harmful Algal Blooms (HABs) in aquatic ecosystems are characterised by the rapid accumulation of algal biomass and/or the production of toxins and harmful metabolites by certain algal species. HAB events may have a broad range of ecological impacts, including, but not limited to, increased mortality of marine organisms (including fish and mammals), detrimental effects on public health and alterations to ecosystem trophic structure [1].

The Red Sea is a Large Marine Ecosystem (LME) and hosts extended coral reef complexes that support high levels of biological diversity and many endemic species. Coral reefs offer vital ecosystem services such as coastal protection, fisheries, tourism and recreation [2–9]. Previous research has demonstrated that HAB events may occur throughout the year in different regions of the Red Sea [10–18]. The reported species during these HAB events include, but not limited to, *Kryptoperidinium foliaceum*, *Noctiluca scintillans/miliaris*, *Heterosigma akashiwo*, *Cochlodinium polykrikoides*, *Ostreopsis sp*. and the cyanobacterium *Trichodesmium erythraeum*. These phytoplankton species were categorized according to the functional group they belong to. For instance, *K. foliaceum*, *N. scintillans/miliaris*, *Ostreopsis sp*., and *C. polykrikoides* belong to dinoflagellates, *H. akashiwo* to raphidophytes and *T. erythraeum* to cyanobacteria. The aforementioned species were mainly responsible for HAB outbreaks previously reported in the Red Sea and have been occasionally associated with severe fish mortalities over the last two decades [11–17].

The first record of the binucleate dinoflagellate *K. foliaceum*, associated with a diatom endosymbiont, was detected in the coastal waters of Al Salif (southern Red Sea) on 8^th^ May 2013 [11]. *N. scintillans/miliaris*, a large heterotrophic dinoflagellate, was detected in the coastal waters of Al Hodeidah, in the southern Red Sea during March 2009 [14]. In the south-central coast of Saudi Arabia, large blooms of *H. akashiwo and Ostreopsis sp*. were observed during May 2010 and February 2012, respectively [15,17]. Winter blooms of the toxic cyanobacteria *T. erythraeum*, which may manifest as “red tides”, were observed off the coast of western Yemen (southern Red Sea) in December 2012 [12].

Previous studies have demonstrated that HABs can be detected and described using satellite remote sensing, which provides bio-optical measurements at spatial and temporal scales not attainable with traditional *in situ* approaches [19,20]. To date, several remote sensing algorithms based on bio-optical and ecological methods have been established to discriminate the aforementioned HABs in the global oceans, including regions adjacent to the Red Sea, such as the Arabian Sea, Indian Ocean and Mediterranean Sea [19–33]. However, to the best of our knowledge no such technique has been utilized to discriminate these HABs in the Red Sea basin. In this study, we developed a remote sensing model that applies satellite and *in situ* datasets to a second-order derivative technique to examine the capability of HAB detection in the Red Sea using a remote sensing algorithm. We apply our method on MODIS-Aqua satellite observations and validate our results using field data collected during several sampling HAB campaigns in the Red Sea over the last two decades.

## Materials and methods

### Satellite data

MODIS-Aqua Level 1A (L1A, calibrated spectral radiance, 1km^2^ resolution) data were obtained from the NASA Goddard Space Flight Centre’s ocean color data archive system (https://oceancolor.gsfc.nasa.gov/). Daily satellite observations were selected to correspond with time periods when blooms of *K. foliaceum*, *N. scintillans/miliaris*, *H. akashiwo*, *Ostreopsis sp*., *T. erythraeum* and *C. polykrikoides* have been observed during field programs in the Red Sea [10–15]. MODIS-Aqua datasets, corresponding to the time periods where previous *in situ* data are available, were acquired to: a) train the model, and b) validate the model results (Table 1). The satellite data were processed to Level 2 using the new atmospheric correction algorithm incorporated in the SeaDAS software (version 7.5) [19,34]. Data products that were extracted from the Level 2 MODIS-Aqua files included remote sensing reflectance (*R*_*rs*_) (for wavelengths 412, 443, 488, 531, 547, 667, 678, and 748 nm), and the algal bloom index (ABI) derived *Chl-a* concentration. The algal bloom index (ABI) algorithm uses normalized water-leaving radiance (*nL_w_*) ratios as a key input parameter to estimate *Chl-a* values associated with HABs, and distinguish algal particles from other non-algal particles in optically complex coastal waters [35,36]. We note that remotely sensed *Chl-a* observations may be hindered in shallow optically complex case II waters where non-phytoplankton substances such as suspended sediments, particulate matter and/or colored dissolved organic matter (CDOM) do not covary in a predictable manner with *Chl-a* [8]. However, validation studies based on a suite of univariate statistical tests have shown a reasonable agreement between remotely sensed measurements and independent *in situ* *Chl-a* data in the Red Sea [37,38]. We are thus confident in the use of satellite-derived *Chl-a* concentrations for supporting the investigation of HABs in the Red Sea.

**Table 1:** Summary of the satellite datasets for the different types of harmful algal blooms (HABs) in the Red Sea.

Name of the harmful algal bloom	Training datasets	Validation datasets
*Kryptoperidinium foliaceum*	26^th^ May 2013 and 2^nd^ June 2013	8^th^ May 2013
*Noctiluca scintillans/ miliaris*	7^th^ February 2004, 21^st^ February 2004, 14^th^ March 2004 and 4^th^ March 2009	3^rd^ March 2009
*Heterosigma akashiwo*	3^rd^ and 10^th^ June 2010	27^th^ May 2010
*Ostreopsis sp*.	16^th^ May 2012, 25^th^ February 2013 and 6^th^ March 2013	27^th^ February 2012
*Trichodesmium erythraeum*	2^nd^ December 2012 and 16^th^ December 2012.	27^th^ December 2012
*Cochlodinium polykrikoides*	23^rd^ November 2008 and 23^rd^ December 2008	13^th^ March 2010

### *In situ* data

**Fig 1:**
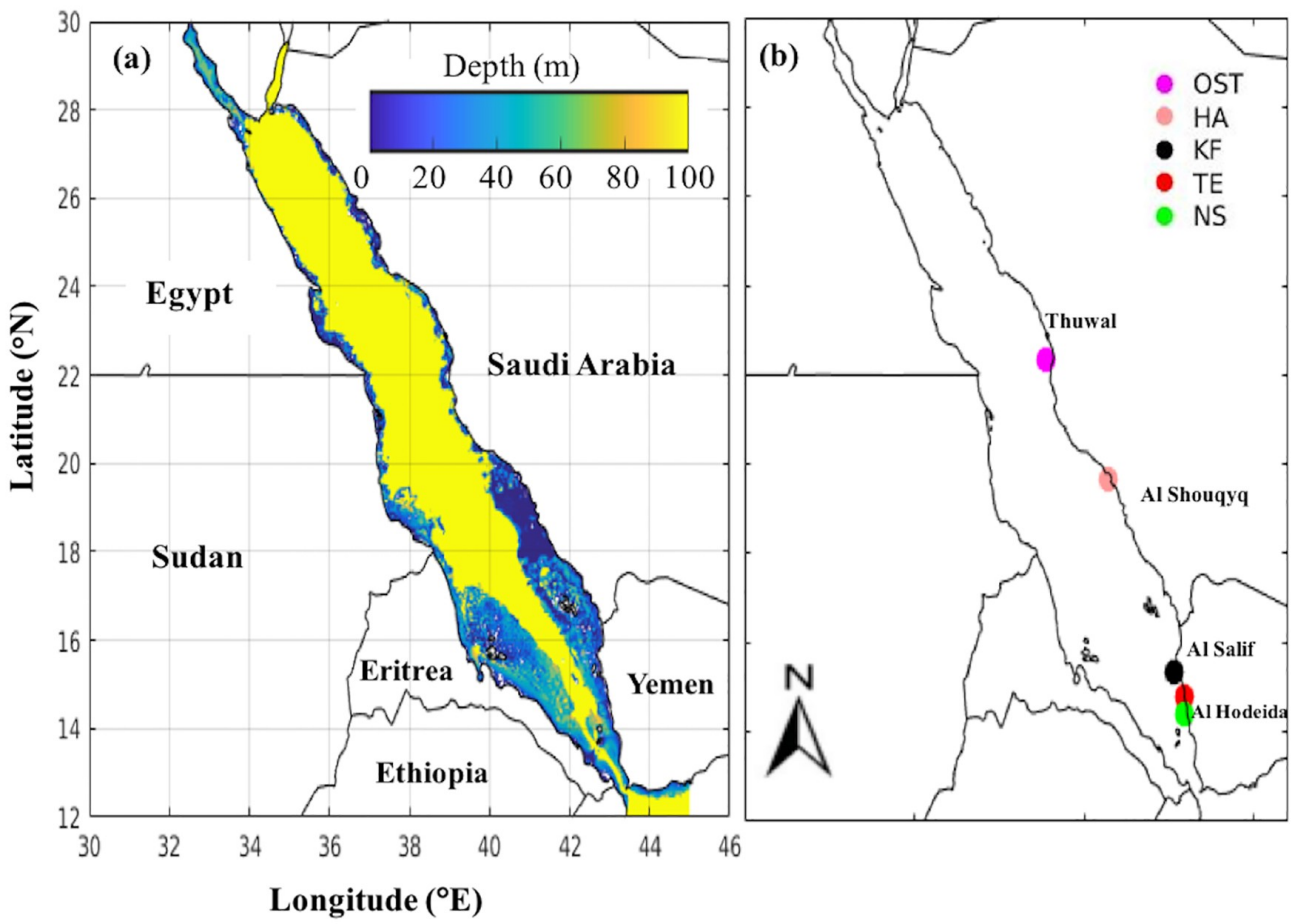
Bathymetry and the position of reported harmful algal blooms (HABs) in the Red Sea. **(a)** Bathymetry of the Red Sea (acquired from the General Bathymetric Chart of the Oceans (GEBCO_2014 Grid, version 20150318, http://www.gebco.net)). **(b)** Map showing the *in situ* sampling locations associated with the different Harmful Algal Blooms (HABs) in the Red Sea; Ost: *Ostreopsis sp*.; HA: *H*. *akashiwo*; KF: *K*. *foliaceum*; TE: *T*. *erythraeum*; NS: *N*. *scintillans/miliaris* (We note that no *in situ* data were collected during the occurrence of *C. polykrikoides* blooms in the Red Sea, although their cyst life-stages were identified along the Red Sea, off the south-western coast of Saudi Arabia [16]).

Surface water samples were collected in the Red Sea during four different field programs. During the field program in November 2012 –August 2013, the dinoflagellate *K. foliaceum* and the cyanobacterium *T. erythraeum* were reported in the coastal waters of Al Salif and the coast of Al Hodeida City, in the southern Red Sea, respectively [11,12]. The field sampling program conducted in the fisheries landing center at Al Hodeida reported an intense bloom of *N. scintillans/miliaris* during March 2009 [14]. The *in situ* study conducted in the Red Sea off the Al Shouqyq coastline (southern Saudi Arabia) on 27^th^ May 2010 reported a water discoloration caused by *H. akashiwo* blooms [17]. Catania et al. [15] reported the occurrence of benthic dinoflagellates *Ostreopsis sp*. on 27 February 2012 at a location off the coasts of Thuwal, Saudi Arabia (Fig 1b and Table 2). The oceanographic measurements that were recorded from the aforementioned field programs included temperature, salinity, and cell counts. The phytoplankton cells were identified and counted using a Sedgwick-Rafter plankton counting chamber. Cells were enumerated and expressed as cells L^-1^. Spatial matchups between the *in situ* measurements and satellite-derived HAB observations were acquired by locating the closest 1 km pixel (nearest latitude and longitude) to the *in situ* sampling location.

**Table 2:** Summary of the different types of harmful algal blooms (HABs) reported by various studies in the Red Sea.

Name of the harmful algal bloom		Date	Cell abundance (L^-1^)	Sampling Location	No. water samples collected	References
Ost	*Ostreopsis sp*.	27^th^ February 2012	12×10^4^	Coasts of Thuwal, Saudi Arabia (22° 19.630′ N, 38° 51.440′ E)	5	[15]
HA	*Heterosigma akashiwo*	27^th^ May 2010	11.4×10^6^	Al Shouqyq city on the southern Red Sea coasts of Saudi Arabia (19°41ʹ05ʺ N, 40°43’20ʺ E)	2	[17]
KF	*Kryptoperidinium foliaceum*	8^th^ May 2013	2.26×10^6^	Coastal water of the Al Salif area, southern Red Sea (15°19ʹ8.96ʺ N, 42°40ʹ49.86ʺ E)	4	[11]
TE	*Trichodesmium erythraeum*	27^th^ December 2012	1.65×10^4^	Coast of Al Hodeida City (14° 47′ 07" N, 42° 56′ 46.31" E)	4	[12]
NS	*Noctiluca scintillans/ miliaris*	3^rd^ March 2009	5.5×10^4^	Coastal waters of the Yemeni Red Sea (14°22ʹ26ʺ N, 42°56’27ʺ E)	5	[14]

### Approach

**Fig 2:**
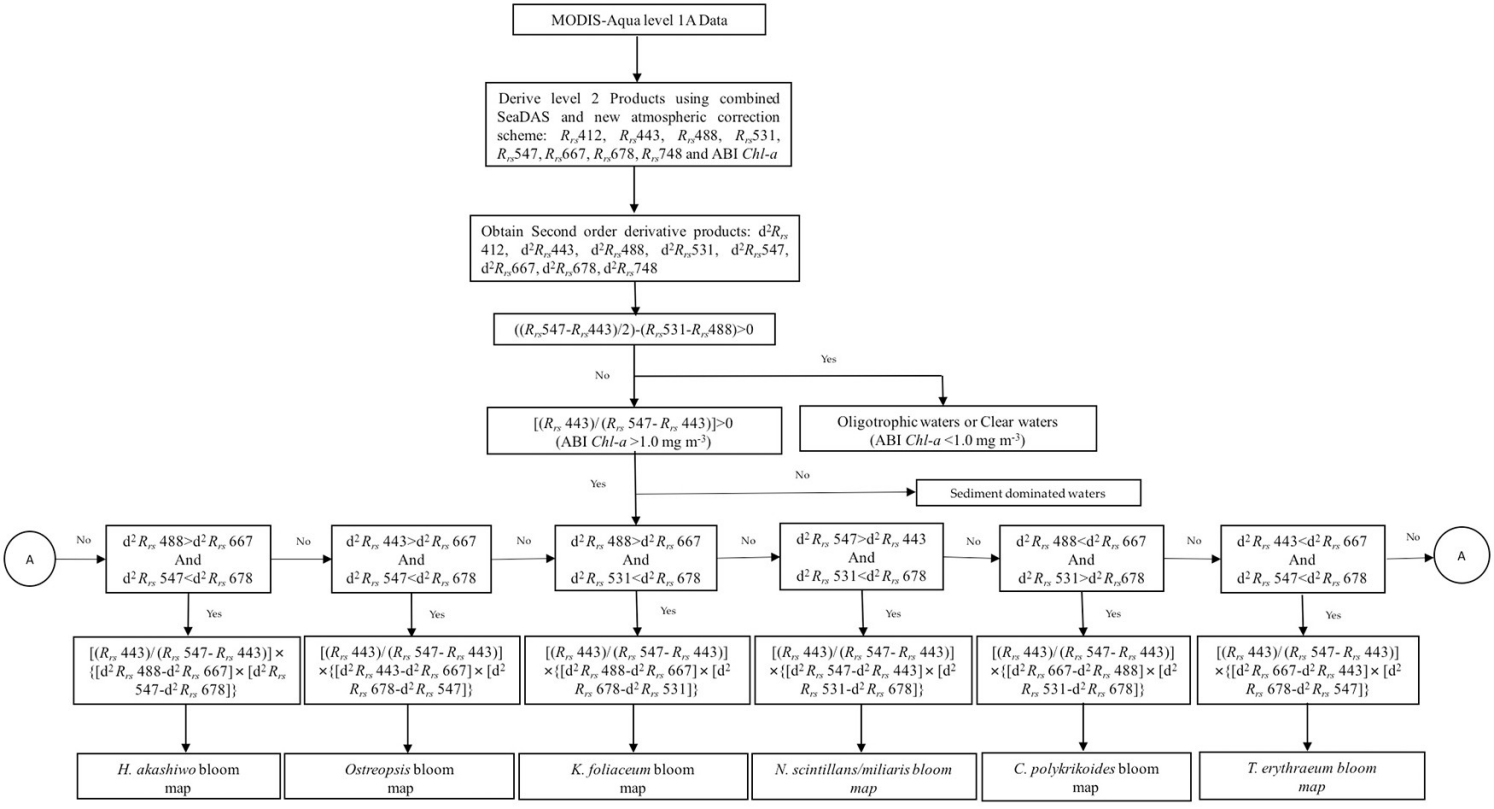
Summary of the steps involved in the development of our model. Flow chart showing the major steps involved in the detection and classification of different Harmful Algal Blooms (HABs) in the Red Sea.

Our modeling approach is comprised of two parts: 1) Deriving *R*_*rs*_ from daily MODIS-Aqua data for the different HAB events that were previously reported in the Red sea waters, and 2) Developing an algorithm based on a derivative analysis to detect species-specific HABs in near-surface waters. Fig 2 highlights the major steps involved in the development and application of the model.

### Training data and spectral analysis

**Fig 3:**
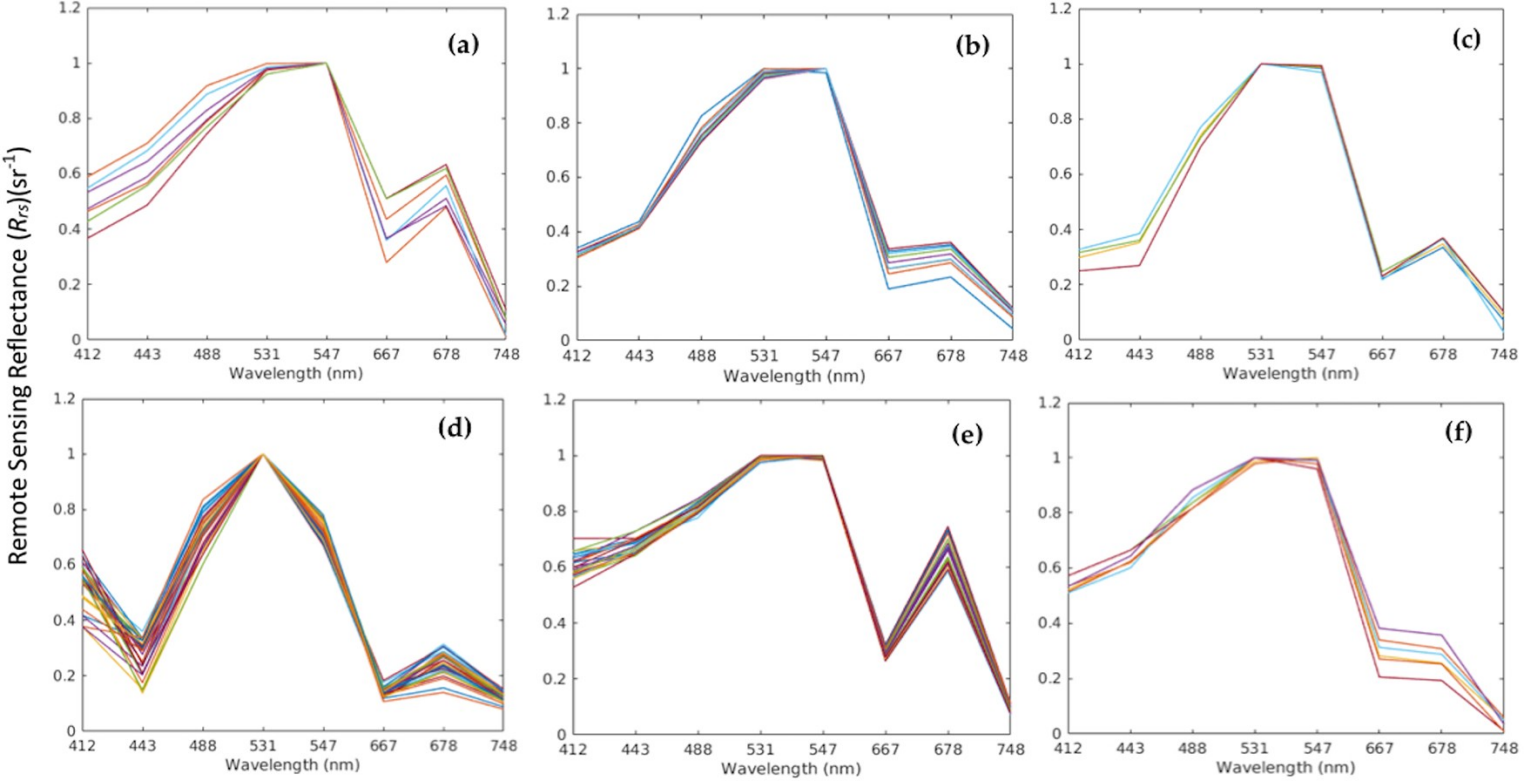
Examples of *R*_*rs*_ derived from daily MODIS-Aqua data for the different HAB species in the Red Sea waters. **(a)** *H*. *akashiwo* **(b)** *Ostreopsis sp*. **(c)** *K*. *foliaceum* **(d)** *N*. *scintillans/miliaris* **(e)** *C. polykrikoides* **(f)** *T*. *erythraeum*.

An independent training dataset was established from *R*_*rs*_ measurements of the different HAB events that were previously reported in the coastal and open waters of the Red Sea. Due to lack of *in situ R*_*rs*_ measurements, a set of training samples were constructed from MODIS-Aqua data concurrent with the *in situ* data. Examples of the *R*_*rs*_ spectra of these HABs in the MODIS-Aqua wavelengths are shown in Fig 3. We note that each HAB species has its unique absorption and backscattering characteristics [19, 39–42] (see S1 and S2 Figs for particulate absorption and backscattering spectra). For instance, the raphidophyte *H. akashiwo* exhibits high *R*_*rs*_ values at 531 and 547 nm, which implies low absorption of pigment concentrations (including fucoxanthin and diadinoxanthin) with high backscattering coefficients at these wavelengths. The dinoflagellate *Ostreopsis sp*. has a significant *R*_*rs*_ peak at 547 nm, which is due to the minimum absorption by *Chl-a* and xanthophyll pigments (diadinoxanthin and peridinin) with, in consequence, backscattering by cells remaining the main factor governing *R*_*rs*_ spectra (Fig 3b) [42]. Moldrup and Garm [40] demonstrated that *K. foliaceum* is characterised by an *R*_*rs*_ peak in the green wavelengths due to the low absorption rate of photopigments associated with this species, including *Chl-a* and c, fucoxanthin, diadinoxanthin, β-carotene, β-zeacarotene and γ-carotene (Fig 3c). The *R*_*rs*_ spectra of the dinoflagellate *N. scintillans/miliaris* exhibits two distinct peaks: one at ~ 531 nm and the other at ~ 678 nm (Fig 3d) [41]. The first peak is due to low absorption and high backscattering caused by a green endosymbiont, whilst the latter is due to the combined effect of reduced absorption and enhanced backscattering [19,41]. In Fig 3e, the strong reflectance peak for *C. polykrikoides* at ~ 547 nm reflects absorption related to the presence of carotenoids [19,22]. As reported in previous studies [19,22,43], the cyanobacterium *T. erythraeum* contains both *Chl-a* and biliproteins (allophycocyanins, phycocyanins and phycoerythrins), which have unique characteristic absorption spectra. The peaks at 531 and 547 nm are due to high backscattering of these biliprotein pigments (Fig 3f). The prominent secondary peak in the far-red region of *R*_*rs*_ spectra for all of the aforementioned species (~ 678 nm) is caused by solar-stimulated *Chl-a* fluorescence (Fig 3a–3f).

### Derivative analysis

Detection of species-specific HABs in the Red Sea depends on the ability to distinguish the unique optical properties associated with each species. Previous studies have demonstrated that band-ratio/difference algorithms can be effectively utilized for mapping HAB events [44,45]. The most commonly used band-ratio/difference algorithms are based on remote sensing reflectance in the blue and green bands (i.e., *R*_*rs*_ 443 nm and *R*_*rs*_ 547 nm) [46,47]. Besides the combination of band-ratio and band-difference algorithms, several other methods have also been used for mapping HABs. One popular technique is the derivative spectra method [48,49]. This method magnifies spectral inflections and enables the detection of small spectral variations. Thus, it can be utilised for the close examination of *R*_*rs*_ spectral patterns. By amplifying *R*_*rs*_ spectral shapes, it highlights features present in the spectra and does not add information that is not already contained in the *R*_*rs*_ spectra [50]. The *R*_*rs*_ spectral shapes of phytoplankton species were examined by calculating the second-order derivative and identifying local maxima (peaks) and minima (troughs) of the *R*_*rs*_ spectrum. The second derivative of *R*_*rs*_ (d^2^ *R*_*rs*_ (λ)) is numerically evaluated as follows:

$$\frac{d^{2}R_{rs}(\text{λ})}{{d\lambda }^{2}}=\frac{R_{rs}(\lambda_{i+1})-2R_{rs}(\lambda_{i})+R_{rs}(\lambda_{i-1})}{{\Delta \lambda }^{2}}$$ (1)

where Δ*λ* = (*λi* − *λ*_*i*+*1*_) is the finite band resolution.

**Fig 4:**
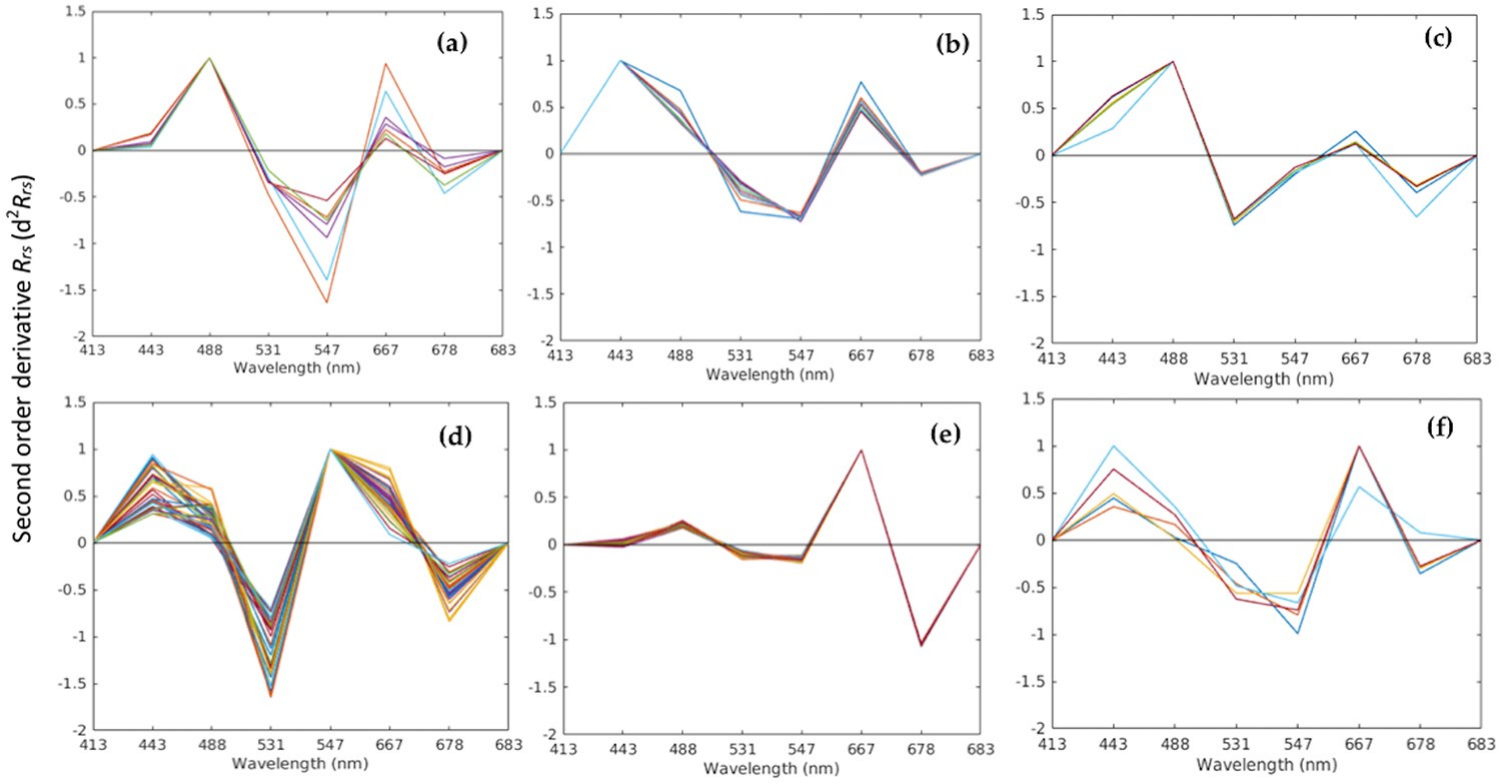
Examples of second-order derivative *R*_*rs*_ from daily MODIS-Aqua data for the different HABs in the Red Sea waters. **(a)** *H*. *akashiwo* **(b)** *Ostreopsis sp*. **(c)** *K*. *foliaceum* **(d)** *N*. *scintillans/miliaris* **(e)** *C. polykrikoides* **(f)** *T*. *erythraeum*.

The current remote sensing model combines both band-ratio/difference algorithms, and second-order derivative analysis, to detect the presence/absence and map the spatial distribution of HAB species. The second derivative of MODIS-Aqua derived *R*_*rs*_ was computed and plotted as a function of wavelength for different HAB species (Fig 4). We note that each HAB species has its unique derivative *R*_*rs*_ spectral signatures. For instance, the derivative *R*_*rs*_ spectra of *H*. *akashiwo* blooms exhibits two prominent peaks (~ 488 nm and ~ 667 nm) and two negative troughs (~ 547 nm and ~ 678 nm) (Fig 4a). The dinoflagellate *Ostreopsis sp*. has two maximum peaks (~ 443 nm and ~ 667nm) and two negative troughs (~547 and ~ 678 nm) of the derivative *R*_*rs*_ spectra (Fig 4b). Two negative troughs at 531 nm and 678 nm are present in the derivative spectra of *K. foliaceum* along with a maximum peak at 488 nm (Fig 4c). The derivative *R*_*rs*_ spectra of *N. scintillans/miliaris* exhibits two positive peaks (~ 443 nm and 547 nm) and two negative troughs (~ 531 nm and ~ 678 nm) (Fig 4d). In the derivative spectra of *C. polykrikoides*, a positive maximum peak is observed at 667 nm and a negative trough at 678 nm (Fig 4e). In addition, *T*. *erythraeum* exhibits two maximum peaks at 443 nm and 667 nm along with two minimum troughs (at ~ 547 nm and 678 nm, Fig 4f). Based on the aforementioned local maxima (peaks) and minima (troughs) of d^2^ *R*_*rs*_ in the entire visible spectrum, we derived different equations (referred to as ‘nequation’ in Table 3) by combining the blue-green band-ratio/difference algorithm and differences in the derivative spectra, to distinguish various HAB species.

**Table 3:** Different sets of nequations for each HAB species based on the band-ratios/difference algorithm and difference in their derivative spectra.

Name of the harmful algal blooms	Nequations
*H. akashiwo*	$\left[\frac{\left(R_{rs}443\right)}{\left(R_{rs}547-R_{rs}443\right)}\right]\times \{[d^{2}R_{rs}488-d^{2}R_{rs}667]\times \left[d^{2}R_{rs}547-d^{2}R_{rs}678\right]\}$
*Ostreopsis sp*.	$\left[\frac{\left(R_{rs}\;443\right)}{\left(R_{rs}\;547-R_{rs}\;443\right)}]\times \{\left[d^{2}R_{rs}443-d^{2}R_{rs}667]\times [d^{2}R_{rs}678-d^{2}R_{rs}547\right]\right\}$
*K. foliaceum*	$\left[\frac{\left(R_{rs}\;443\right)}{\left(R_{rs}\;547-R_{rs}\;443\right)}]\times \{\left[d^{2}R_{rs}488-d^{2}R_{rs}667]\times [d^{2}R_{rs}678-d^{2}R_{rs}531\right]\right\}$
*N. scintillans/miliaris*	$\left[\frac{\left(R_{rs}\;443\right)}{\left(R_{rs}\;547-R_{rs}\;443\right)}]\times \{\left[d^{2}R_{rs}547-d^{2}R_{rs}443]\times [d^{2}R_{rs}531-d^{2}R_{rs}678\right]\right\}$
*C. polykrikoides*	$\left[\frac{\left(R_{rs}\;443\right)}{\left(R_{rs}\;547-R_{rs}\;443\right)}]\times \{\left[d^{2}R_{rs}667-d^{2}R_{rs}488]\times [d^{2}R_{rs}531-d^{2}R_{rs}678\right]\right\}$
*T. erythraeum*	$\left[\frac{\left(R_{rs}\;443\right)}{\left(R_{rs}\;547-R_{rs}\;443\right)}]\times \{\left[d^{2}R_{rs}667-d^{2}R_{rs}443]\times [d^{2}R_{rs}678-d^{2}R_{rs}547\right]\right\}$

### Remote sensing parameters for monitoring HABs in the Red Sea

Several different parameters have been used to investigate occurrence of HABs, including false color composite imagery, satellite-derived *Chl-a* maps, and spectral analysis-based bloom maps [51,52]. These parameters have enabled us to detect the presence/absence of HABs and map the spatial extent of different HAB species that were previously reported by various field programs in the Red Sea. A detailed synopsis of the HAB analysis is provided as follows.

The first step of this analysis was to visually interpret the abnormality of ocean color in the Red Sea during HAB events. To achieve this, we processed false-color composite MODIS images by combining *R*_*rs*_ in the NIR, green and blue wavelength bands (also known as the RGB band). Hu et al. [53] demonstrated that false color imagery can easily distinguish between dark features caused by high absorption of light related to the presence of *Chl-a*, and bright features caused by non-pigment materials such as sediment, corals and shallow bathymetry. Secondly, to investigate *Chl-a* concentrations during a HAB event, we examined satellite-derived *Chl-a* generated using the ABI algorithm. Gokul and Shanmugam [19] demonstrated that satellite-derived ABI *Chl-a* can effectively estimate *Chl-a* values associated with HABs, and discriminate algal bloom patches from other non-algal particles in optically complex coastal waters. In addition, based on the satellite derived *R*_*rs*_ spectra (training dataset) and its second-order derivatives of different phytoplankton functional types (PFTs), we produced bloom maps in order to detect the presence/absence and map the spatial distribution of different phytoplankton species that were known for HAB outbreaks in the Red Sea (see Fig 2). Finally, *in situ* measurements of the different phytoplankton cell counts (related to these HAB species) were used for the validation of the model results.

## Results

**Fig 5:**
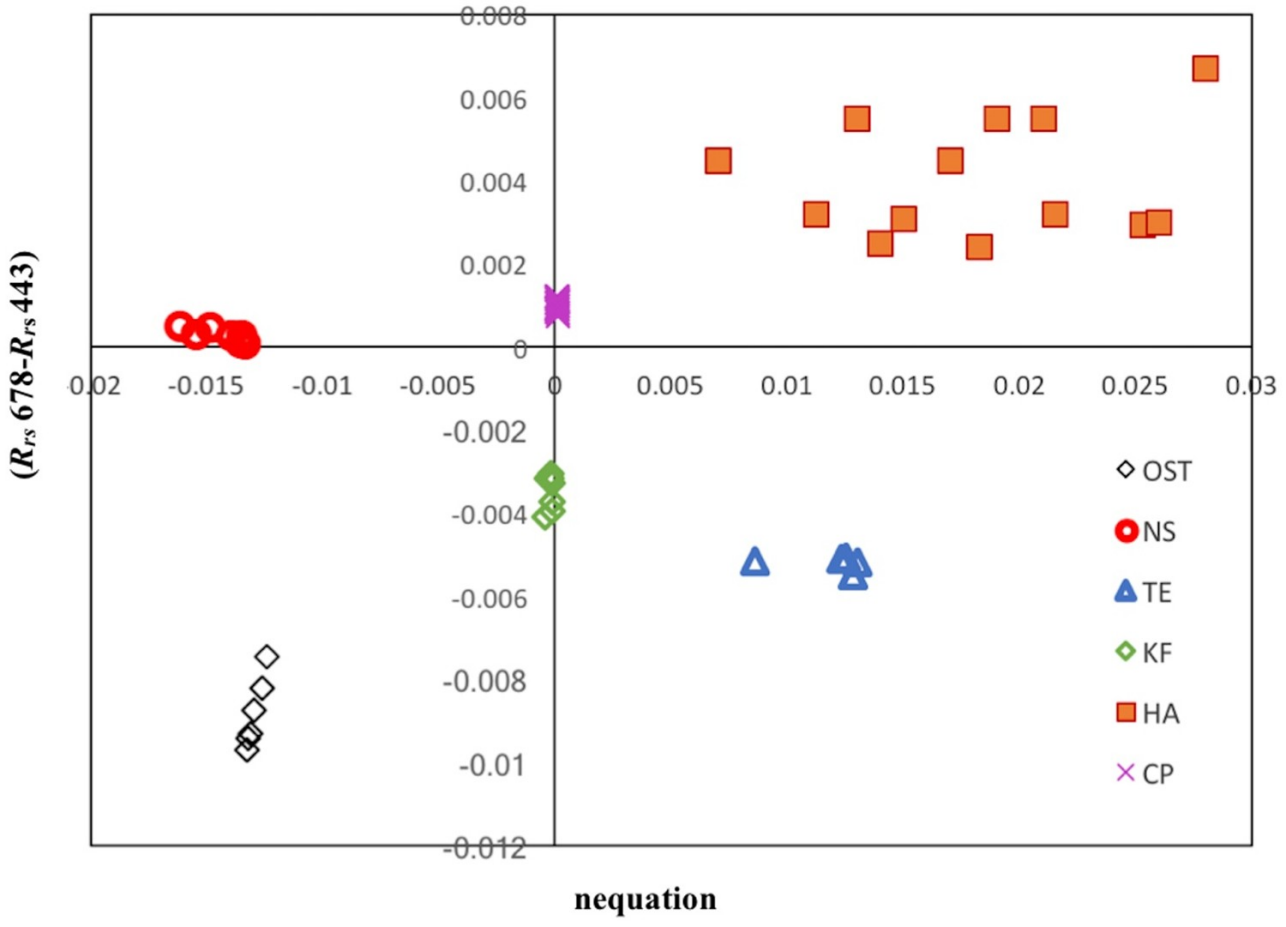
Distinct cluster of different HAB species (based on training datasets) in the Red Sea waters. Ost- *Ostreopsis sp*.; NS- *N*. *scintillans/miliaris*; TE- *T*. *erythraeum*; KF-*K*. *foliaceum*; HA-*H*. *akashiwo*; CP- *C. polykrikoides*.

The proposed remote sensing algorithm was applied to daily MODIS images that were selected during HAB events previously reported in the coastal and open waters of the Red sea (see Materials and methods). A scatterplot of the output from nequations versus the *R*_*rs*_ difference (*R*_*rs*_ 678−*R*_*rs*_ 443) is shown in Fig 5. Based on the derivative analysis, all these types of HABs were shown to be well clustered and distinguishable. For convenience, we categorized these different types of HAB species according to their regional locations in the Red Sea. For instance, *Ostreopsis sp*. and *H. akashiwo* blooms were detected in the central Red Sea, whilst the remaining blooms (*K. foliaceum*, *N. scintillans/miliaris*, *T. erythraeum* and *C. polykrikoides*) occurred in the southern Red Sea. The spatial distributions of these HAB events in the central and southern Red Sea are discussed in the following section.

### Regional examples

#### Central Red Sea

**Fig 6:**
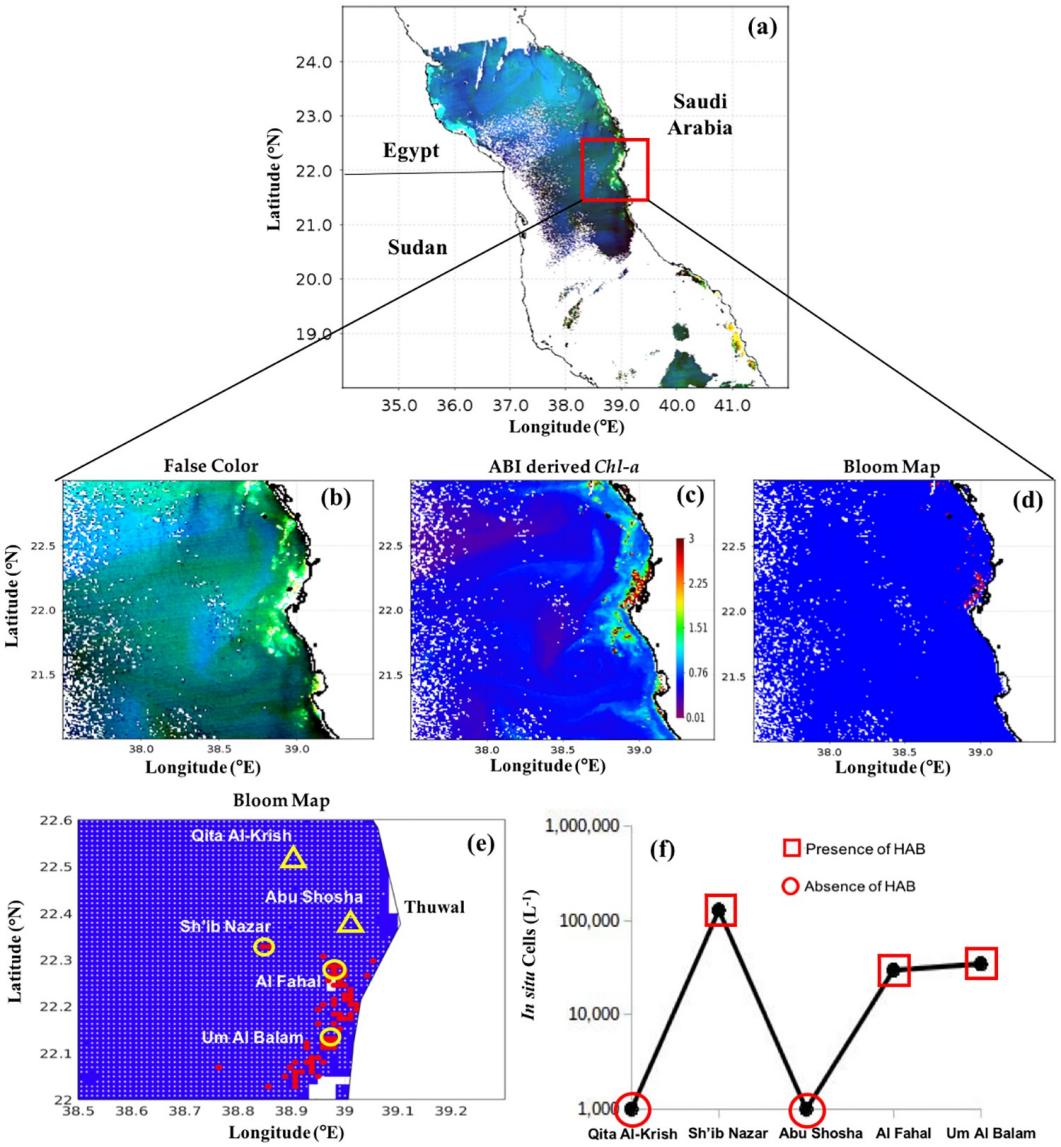
Remotely sensing *Ostreopsis sp*. blooms in the central Red Sea and comparison with *in situ* measurements. **(a)** and **(b)** False color composite image generated using the NIR, green and blue reflectances (i.e., R = *R*_*rs*_ (748), G = *R*_*rs*_ (547), B = *R*_*rs*_ (443)). **(c)** Satellite-derived *Chl-a* using the ABI algorithm. **(d)** and **(e)** MODIS-Aqua-derived maps of the *Ostreopsis sp*. blooms in the central Red Sea on 27^th^ February 2012 [In **(e)** the red and white dots denote the presence and absence of HABs, respectively, as detected using satellite data. Also, the yellow circles and yellow triangles indicate the *in situ* sample points and highlight the presence and absence of *Ostreopsis sp*. blooms, respectively]. **(f)** Variations in *Ostreopsis sp*. cell counts recorded from the different sampling sites in the central Red Sea on 27^th^ February 2012 [red squares and red circles denote satellite detection of HAB presence and absence, respectively].

**Fig 7:**
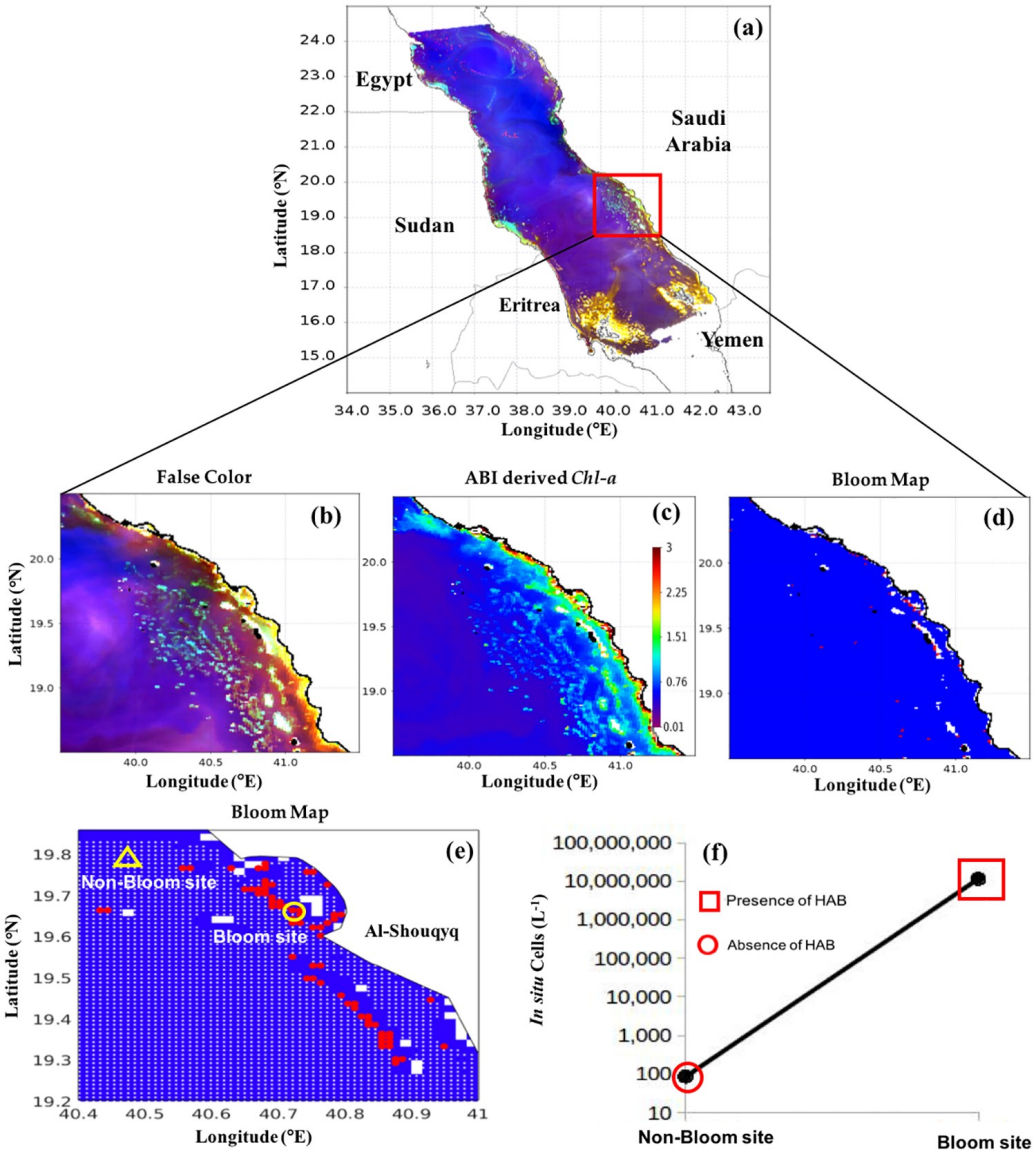
Remotely sensing *H*. *akashiwo* blooms in the central Red Sea and comparison with *in situ* measurements. **(a)** and **(b)** False color composite image generated using the NIR, green and blue reflectances (i.e., R = *R*_*rs*_ (748), G = *R*_*rs*_ (547), B = *R*_*rs*_ (443)). **(c)** Satellite-derived *Chl-a* using ABI algorithm. **(d)** and **(e)** MODIS-Aqua-derived maps of the *H*. *akashiwo* blooms in the Red Sea on 27^th^ May 2010 [In **(e)** the red and white dots denote the presence and absence of HABs, respectively, as detected using satellite data. Also, the yellow circle and yellow triangle indicates the *in situ* sample points and highlight the presence and absence of *H*. *akashiwo* blooms, respectively]. **(f)** Variations in *H*. *akashiwo* cell counts recorded from the two different sampling sites in the Red Sea off the southern coast of Saudi Arabia on 27^th^ May 2010 [red square and red circle denotes the satellite detection of HAB presence and absence, respectively].

To detect blooms of *Ostreopsis sp*. and *H. akashiwo*, we utilised high-resolution MODIS-Aqua images taken on 27^th^ February 2012 and 27^th^ May 2010 respectively, over the central Red Sea, along the south-central coast of Saudi Arabia. Figs 6 and 7 illustrate how the presence of these two blooms may be revealed by false color imagery, satellite-derived *Chl-a* and bloom maps generated using the remote sensing model. For instance, the bright features observed in Figs 6 and 7b were probably caused by suspended sediments or the presence of coral reefs. Such features limit the utility of false color imagery for the detection of water discoloration caused by HABs. However, high *Chl-a* concentrations (up to 3 mg m^-3^) occurred in the same areas (clearly seen as red features in the Figs 6 and 7c). Thus, in comparison to the false color imagery, ABI-derived values of *Chl-a* appear to be less sensitive to the presence of substances other than phytoplankton, thereby reducing the possibility that pixels characterised by non-algal substances being confused with those that are representative of *Ostreopsis sp*. and *H. akashiwo* blooms. Based on the current model, the presence of these two blooms (red patches in of Figs 6, 7d and 7e) appeared to coincide spatially with high ABI-derived *Chl-a* concentrations (Figs 6 and 7c), indicating that the model was able to detect the presence/absence of these blooms. The presence of *Ostreopsis sp*. and *H*. *akashiwo* blooms detected by the remote sensing model were in agreement with maximum *in situ* cell counts recorded along the south-central coast of Saudi Arabia during 27^th^ February 2012 and 27^th^ May 2010 (Figs 6 and 7f and Table 3), respectively.

#### Southern Red Sea

**Fig 8:**
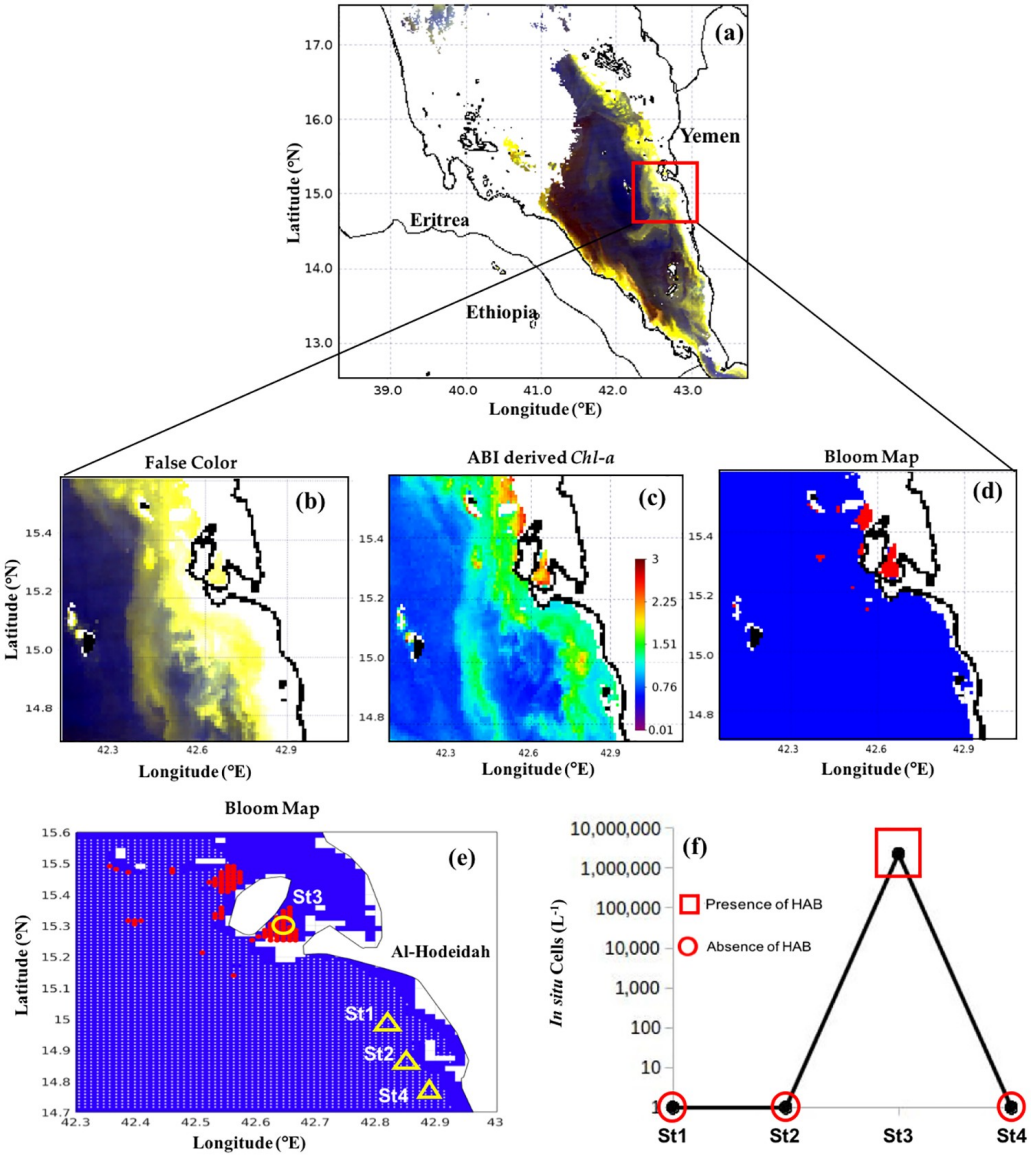
Remotely sensing *K. foliaceum* blooms in the southern Red Sea and comparison with *in situ* measurements. **(a)** and **(b)** False color composite image generated using the NIR, green and blue reflectances (i.e., R = *R*_*rs*_ (748), G = *R*_*rs*_ (547), B = *R*_*rs*_ (443)). **(c)** Satellite-derived *Chl-a* using ABI algorithm. **(d)** and **(e)** MODIS-Aqua-derived maps of the *K. foliaceum* blooms in the Red Sea on 8^th^ May 2013 [In **(e)** the red and white dots denote the presence and absence of HABs, respectively, as detected using satellite data. Also, the yellow circle and yellow triangles indicate the *in situ* sample points and highlight the presence and absence of *K. foliaceum* blooms, respectively]. **(f)** Variations in *K. foliaceum* cell counts recorded from the four different sampling stations in the coastal waters of Al Salif, southern Red Sea on 8^th^ May 2013 [red squares and red circles denote satellite detection of HAB presence and absence, respectively].

**Fig 9:**
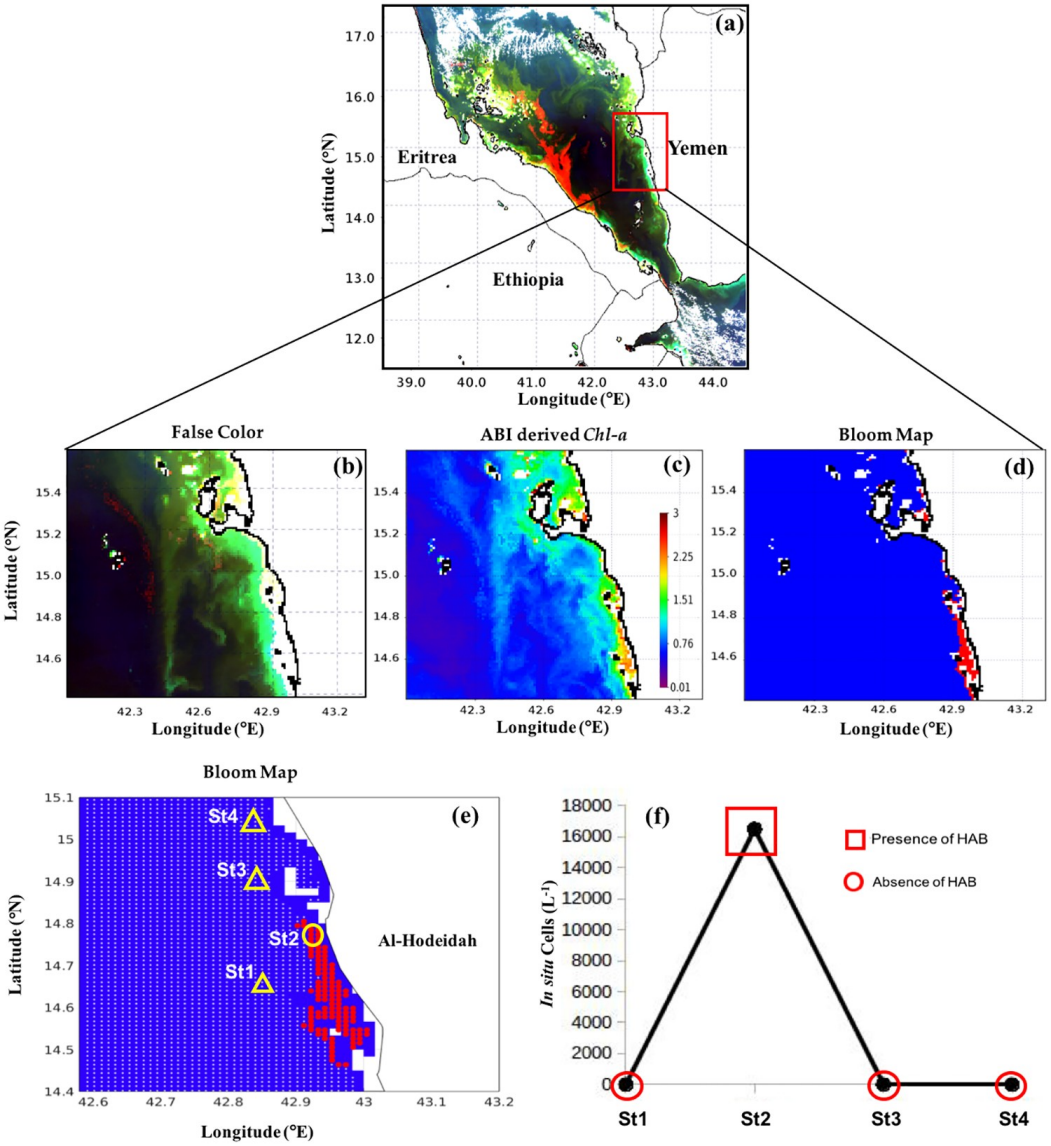
Remotely sensing *T*. *erythraeum* blooms in the southern Red Sea and comparison with *in situ* measurements. **(a)** and **(b)** False color composite image generated using the NIR, green and blue reflectances (i.e., R = *R*_*rs*_ (748), G = *R*_*rs*_ (547), B = *R*_*rs*_ (443)). **(c)** Satellite-derived *Chl-a* using ABI algorithm. **(d)** and **(e)** MODIS-Aqua-derived maps of the *T*. *erythraeum* blooms in the Red Sea on 27^th^ December 2012 [In **(e)** the red and white dots denote the presence and absence of HABs, respectively, as detected using satellite data. Also, the yellow circle and yellow triangles indicate the *in situ* sample points and highlight the presence and absence of *T*. *erythraeum* blooms, respectively]. **(f)** Variations in *T*. *erythraeum* cell counts recorded from the four different sampling stations in the Yemeni coastal waters off Red Sea, near Al Hodeida City on 27^th^ December 2102 [red squares and red circles denote satellite detection of HAB presence and absence, respectively].

**Fig 10:**
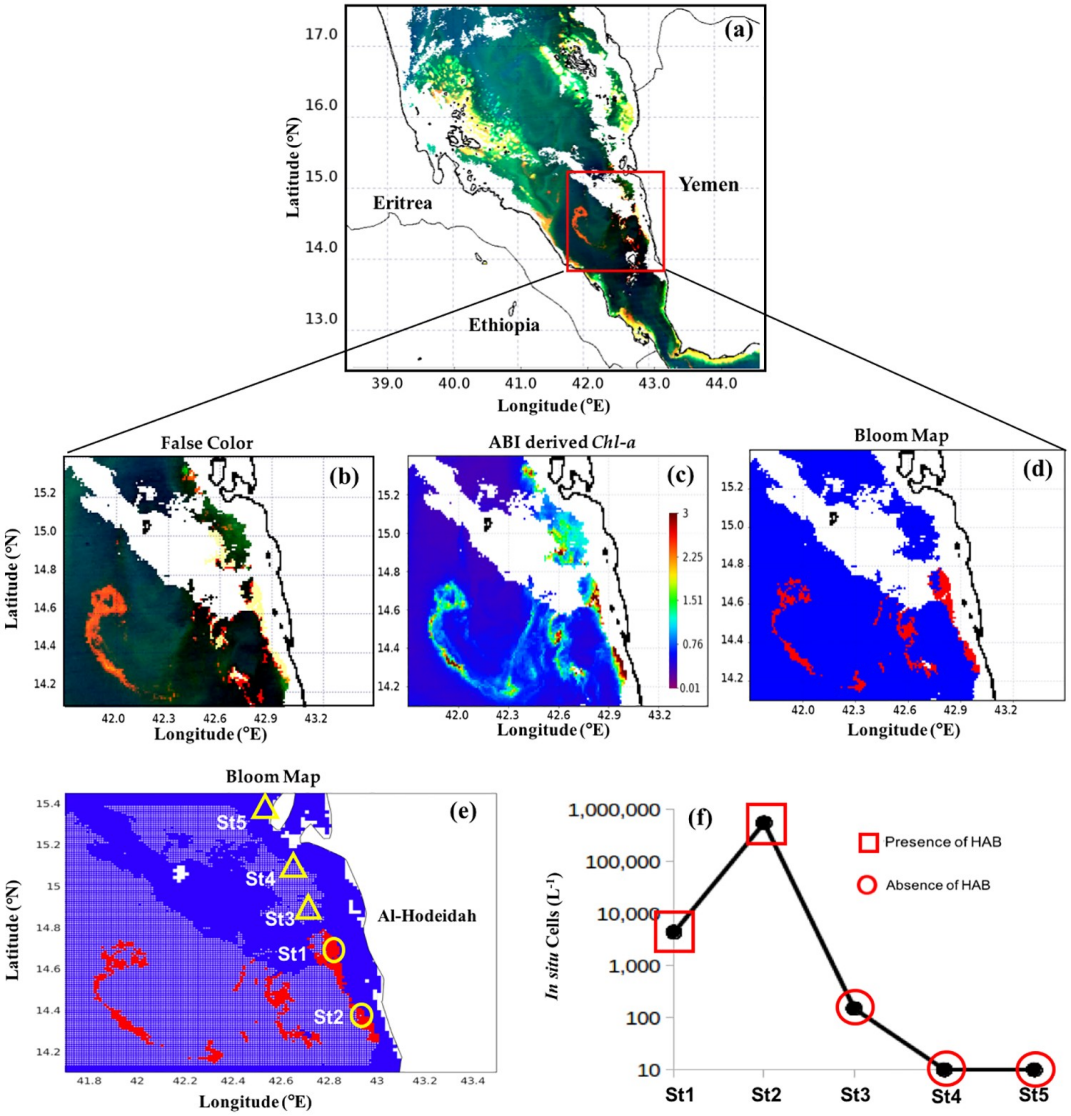
Remotely sensing *N*. *scintillans/miliaris* blooms in the southern Red Sea and comparison with *in situ* measurements. **(a)** and **(b)** False color composite image generated using the NIR, green and blue reflectances (i.e., R = *R*_*rs*_ (748), G = *R*_*rs*_ (547), B = *R*_*rs*_ (443)). **(c)** Satellite-derived *Chl-a* using ABI algorithm. **(d)** and **(e)** MODIS-Aqua-derived maps of the *N*. *scintillans/miliaris* blooms in the Red Sea on 3^rd^ March 2009 [In **(e)** the red and white dots denote the presence and absence of HABs, respectively, as detected using satellite data. Also, the yellow circles and yellow triangles indicate the *in situ* sample points and highlight the presence and absence of *N*. *scintillans/miliaris* blooms, respectively]. **(f)** Variations in *N*. *scintillans/miliaris* cell counts recorded from the five different stations in the coastal waters of Yemen, southern Red Sea during 3^rd^ March 2009 [red squares and circles denote satellite detection of HAB presence and absence, respectively].

Similarly, we applied the model to MODIS-Aqua images taken on 8^th^ May 2013 and 27^th^ December 2012 to investigate blooms of *K. foliaceum* and *T. erythraeum*, respectively, in the Yemeni coastal waters of the southern Red Sea. As reported by Alkawri [9,10], these two blooms occurred in the shallow waters, where reflective features (e.g. suspended sediments and the sea bed) may disrupt the signal of these phytoplankton blooms. This hindered the utility of false color composite imagery for the visual differentiation of these blooms from features associated with surrounding non-bloom and sediment dominated waters (Figs 8 and 9b). In contrast, *N. scintillans/miliaris* blooms on 3^rd^ March 2009 were clearly observed as red features in the false color imagery, as a result of enhanced reflectance at red wavelengths (Fig 10b). Also, *Chl-a* associated with these patches was much higher than the surrounding non-bloom waters (Fig 10c). The spatial patterns of *K. foliaceum*, *T. erythraeum* and *N. scintillans/miliaris* blooms, as depicted by their bloom maps, were highly correlated with high *Chl-a* values (> 2 mg m^-3^) in those areas (Figs 8–10c and 10d). Though the field observations were spatially limited, when applied to MODIS-Aqua data, our remote sensing model was able to map the spatial patterns of these HAB species in the Yemeni coastal waters of the southern Red Sea (Figs 8–10d and 10e). The presence of *K. foliaceum*, *T. erythraeum* and *N. scintillans/miliaris* blooms detected by MODIS-Aqua were found to coincide with higher observed *in situ* cell counts that were collected along the Yemeni coastal waters on 8^th^ May 2013, 27^th^ December 2012 and 03^rd^ March 2009 (Figs 8–10f and Table 3), respectively.

To assess the accuracy of the remote sensing model, four measures of accuracy were derived from an error matrix: the user’s accuracy, producer’s accuracy, overall accuracy and kappa coefficient (see definitions and formulations in S1 File). Based on the spatial matchups between *in situ* measurements and satellite-derived HAB observations, we constructed an error matrix to provide an accuracy assessment and error characterization of our model (Table 4). The results of the accuracy assessment for detecting the presence and absence of different HAB species in the Red Sea are outlined in Table 4 (footnote ‘a’). Overall, the results reveal that satellite-derived HAB observations exhibit a good agreement with the *in situ* datasets of individual HAB species.

**Table 4:** Statistical accuracy assessment results for detecting the presence and absence of different HAB species in the Red Sea waters^a^.

		***No*. *of satellite match ups***					
		***Ostreopsis sp*.**	***H. akashiwo***	***K. foliaceum***	***T. erythraeum***	***N. scintillans/miliaris***	**Total**
***No*. *of in situ sampling locations***	***Ostreopsis sp*.**	**5**	**0**	**0**	**0**	**0**	**5**
	***H. akashiwo***	**0**	**2**	**0**	**0**	**0**	**2**
	***K. foliaceum***	**0**	**0**	**4**	**0**	**0**	**4**
	***T. erythraeum***	**0**	**0**	**0**	**4**	**0**	**4**
	***N. scintillans/miliaris***	**0**	**0**	**0**	**1**	**4**	**5**
	**Total**	**5**	**2**	**4**	**5**	**4**	**20**

**^a^Overall accuracy = ((5+2+4+4+4)/20)×100 = 95%. Producer’s Accuracy:** Ostreopsis sp. = (5/5) ×100 = 100%; *H. akashiwo* = (2/2) ×100 = 100%; *K. foliaceum* = (4/4) ×100 = 100%; *T. erythraeum* = (4/5) ×100 = 80%; *N. scintillans/miliaris* = (4/4) ×100 = 100%. **User’s Accuracy**: Ostreopsis sp. = (5/5) ×100 = 100%; *H. akashiwo* = (2/2) ×100 = 100%; *K. foliaceum* = (4/4) ×100 = 100%; *T. erythraeum* = (4/5) ×100 = 100%; *N. scintillans/miliaris* = (4/5) ×100 = 80%. **Kappa coefficient** = (NA-B)/(N^2^-B) = 0.9, where N = 20; A = 19; B = 85.

### Relationships between satellite-derived *Chl-a* and *in situ* phytoplankton cell counts

**Fig 11:**
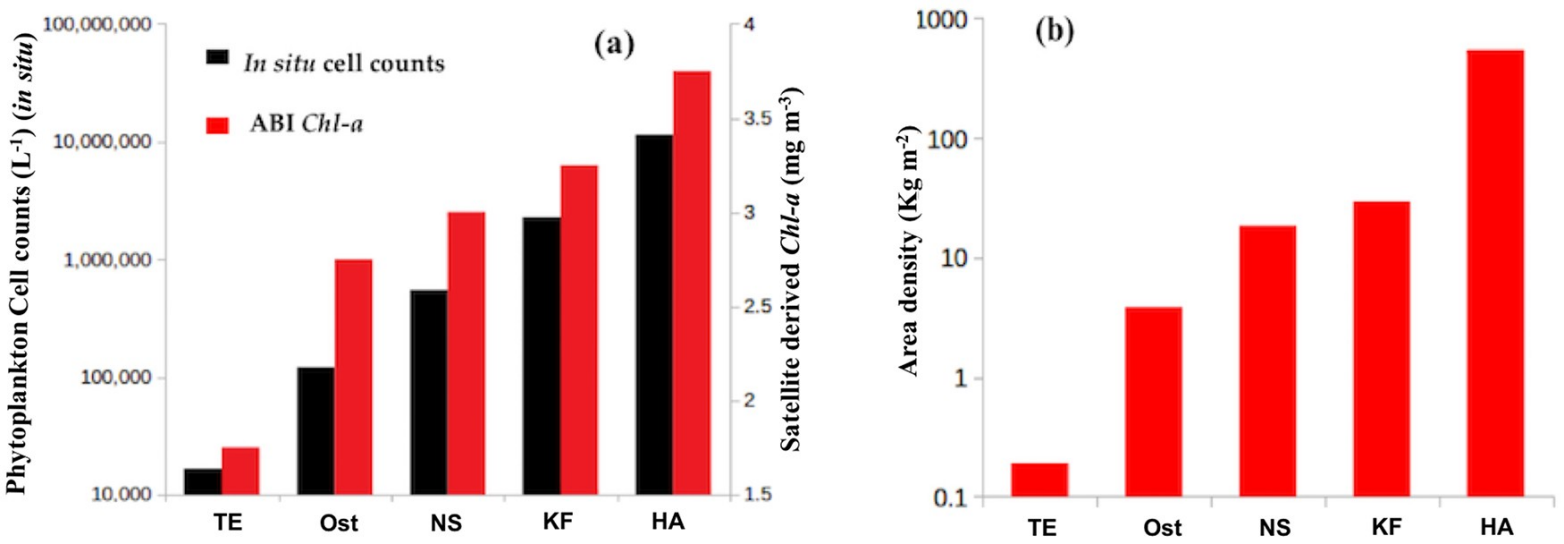
Remotely sensing *Chl-a* concentrations associated with different HAB species in the Red Sea and comparison with *in situ* phytoplankton cell counts. **(a)** Variations in total cell counts of harmful species and their satellite-derived *Chl-a* concentrations observed in the Red Sea. **(b)** Variations in the area density of remotely sensed HABs in the Red Sea. TE- *Trichodesmium erythraeum*; Ost- *Ostreopsis sp*.; NS- *Noctiluca scintillans*; KF-*Kryptoperidinium foliaceum*; HA-*Heterosigma akashiwo*.

In order to assess the performance of the remote sensing model, we compared satellite-derived *Chl-a* and *in situ* phytoplankton cell counts data for each HAB species. As evident in Fig 11a, the *in situ* cell count increases in conjunction with satellite-derived *Chl-a* concentrations for each of the observed HAB species. In addition, we also computed the area density for each HAB species, based on the spatial coverage of bloom pixels (area covered by the HAB event) and their corresponding *in situ* phytoplankton cell counts. Fig 11b shows the variations in the area density of the different HAB species observed in the Red Sea. The area density of *T. erythraeum* was the lowest (0.3 kg m^-2^) amongst all of the species because of their low cell abundance (1.6* 10^4^ L^-1^), whilst the highest was observed for *H. akashiwo* with an area density of 540 kg m^-2^. The second highest area density was attributed to *K. foliaceum* (29 kg m^-2^). The overall area density pattern of HAB species followed the trend *H. akashiwo* > *K. foliaceum* > *N. scintillans/miliaris* > *Ostreopsis sp*. > *T. erythraeum*.

## Discussion

### Accuracy of the present HAB detection model in the Red Sea

Over the last two decades, an increase in the number of HAB events has been observed along the Red Sea coast [11–17]. We note that many of these species have been reported to cause severe fish mortalities and numerous ecological impacts in the region [10–14]. However, as the Red Sea remains relatively unexplored, *in situ* datasets are limited. Alternatively, in parallel with *in situ* measurements, satellite remote sensing can provide important information on the spatial distribution of HABs. Until recently, several bio-optical algorithms have been established to identify the dominant PFTs in the oceans [19–33]. Brewin et al. [27] demonstrated that these algorithms use different PFT definitions and retrieve different variables including, but not limited to, multiple taxonomic groups and phytoplankton size fractions based on *Chl-a* or volume [25]. These PFT algorithms were mainly based on second-order anomalies of *R*_*rs*_ spectra [30], band-ratio *R*_*rs*_ spectra [33], absorption [31] and backscattering spectra [32]. Although the aforementioned algorithms have provided promising results for distinguishing PFTs from space, they have pointed out several limitations, one being their limited ability to distinguish between different HAB species that belong to the same PFT. To overcome this, we developed a model that exploits the spectral characteristics of different HAB species using a second-order derivative approach, along with the *R*_*rs*_ band-ratio/difference method, to detect and classify HABs in the Red Sea. The second-order derivative technique was trained based on the spectral curvature and shape of *R*_*rs*_ data—two factors used to distinguish species-specific HABs from other phytoplankton and non-phytoplankton features in the coastal and open waters of Red Sea. The prominent peaks in the *R*_*rs*_ spectra at green (~ 531 and/or 547nm) and red wavelengths (~ 678 nm) for these HAB species are represented by noticeable troughs in the respective wavelengths for the d^2^ *R*_*rs*_ spectra (see Figs 3 and 4). We note that the spatial patterns of these HABs species, inferred from bloom maps, generated via the remote sensing model, appeared to be consistent with satellite-derived *Chl-a*. Our results show that the current model detects the aforementioned HAB species with adequate accuracy, specifically at high *Chl-a* concentrations (> 1 mg m^-3^). In addition, our model was also capable of mapping the spatial extent of these HAB species in the Red Sea waters. Although HABs mainly occur locally, the dynamic regional circulation can transfer water masses hundreds of kilometers away [9,54] (see Fig 10d and 10e). Combining false color satellite imagery from spectral *R*_*rs*_ data, satellite *Chl-a*, and knowledge of local waters, our study has demonstrated that it is possible to differentiate HABs from the reflective feature associated with suspended sediments, corals and shallow bathymetry.

### Limitations of the present HAB model

In this section, we acknowledge some limitations of the HAB model, identify and summarize the main issues, and wherever possible, suggest solutions. Firstly, the Red Sea is surrounded by deserts and is frequently influenced by dust storms and aerosols, which makes it challenging to apply atmospheric corrections [38]. Since our model utilises satellite ocean color data, the accuracy of the detection and classification of HABs may also depend on the atmospheric correction results (i.e., water leaving radiance (*L_w_*)) [19]. Recent studies have demonstrated that the standard atmospheric correction scheme may result in erroneous and negative *L_w_* across the entire visible wavelengths in optically complex oceanic waters, where dense HABs (e.g. *N. scintillans/miliaris* and *C. polykrikoides*) absorb strongly in the blue wavelengths and reflect at NIR bands [34,55]. Thus, the associated enhanced water signals may interfere with the standard aerosol correction scheme to produce negative *L_w_* (and subsequently *R*_*rs*_) for most of the HAB pixels [20,22,34]. This problem was circumvented by utilising an atmospheric correction algorithm [34] that was mainly developed for optically complex, oceanic waters dominated by suspended sediments and HABs [19]. This atmospheric correction algorithm, developed by Singh and Shanmugam [34], was adopted in the current model because the overestimation of aerosol radiance related to the iterative NIR method (i.e., standard aerosol correction scheme) [56], and its extrapolation to the visible spectrum in sediment-laden and algal bloom waters, leads to negative values of *R*_*rs*_ [34,38]. To overcome these issues, a dimensionless coefficient (κ) was introduced in this atmospheric algorithm to account for the non-zero water leaving radiance contribution in the NIR bands, and the extrapolation of the aerosol radiance to the visible bands [19,34], thereby leading to positive retrievals of *R*_*rs*_ across all visible wavelengths in HAB dominated waters (see Fig 3). This enabled the more accurate detection and discrimination of HABs by the current model, without any pixels being falsely masked out or eliminated by strict quality control criteria [19,34].

Secondly, the southern Red Sea is adversely affected by atmospheric conditions (haze and clouds) during the summer monsoon (June-September), which severely reduces the retrieval of satellite ocean color observations from MODIS-Aqua data [38, 57–60]. This limited the ability of the present remote sensing model to assess *Chl-a* and HABs in the southern Red Sea during summer. For instance, Alkawri et al. [10,12] reported the occurrence of intense blooms of the toxic dinoflagellate *Protoperidinium quinquecorne* (up to 14.3 × 10^6^ L^-1^) and *Pyrodinium bahamense* var. *bahamense* (up to 3.3 × 10^5^ L^-1^) in the Yemeni coastal waters of the southern Red Sea during June 2012 and August 2013, respectively. However, the persistent presence of clouds, sun-glint, and sensor saturation during these months may hinder the ability of the model to detect the presence/absence of these HABs and map their spatial extent in the southern Red Sea. Due to these adverse atmospheric conditions over the Red Sea region, the current model can only utilize a limited number of daily MODIS-Aqua images for mapping the spatial distribution of HAB species in this basin.

There is also another limitation related to the spectral analysis adopted in the present HAB model. For instance, the *R*_*rs*_ spectra of these HAB species (excluding *T. erythraeum*) exhibits two significant peaks in the green and red wavelengths, due to different phytoplankton properties (e.g. absorption by *Chl-a* and other pigments, cell density and other associated optical properties). However, water dominated by cyanobacteria (e.g. *Trichodesmium*) exhibits relatively low values at 678 nm (see Fig 3f). This indicates a less-dense bloom (*Chl-a* concentration < 2 mg m^-3^; see Fig 9c) when compared to the *Trichodesmium* MODIS spectra reported in the global oceans, including regions adjacent to the Red Sea (such as Arabian Sea, Indian Ocean) [19,22,61]. In addition, highly reflective features (e.g. suspended sediments, corals, shallow bathymetry) were found to interfere with the spectral detection of *Trichodesmium* blooms [19,20]. In such conditions, it was hard to optically distinguish *Trichodesmium* blooms from other strong features using satellite ocean colour observations. Existing models require additional information (e.g. SST, wind and depth) to properly distinguish *Trichodesmium* blooms from other phytoplankton and non-phytoplankton features [19]. We also note that the intensity and position of *R*_*rs*_ peaks and spectral characteristics in the far-red wavelengths are influenced by both phytoplankton reflectance and the exponentially increasing absorption of water molecules [62–64]. For instance, the higher the concentration of phytoplankton and/or nearer to surface water is, the stronger the *R*_*rs*_ peak. It is noted that for higher *Chl-a* concentrations (> 2 mg m^-3^), these HABs form surface scum and the corresponding *R*_*rs*_ around 678 nm are enhanced [19,39–44]. Also, the peak in the red wavelength (around 678 nm) can be shifted into the NIR region (700–720 nm) at much higher *Chl-a* concentrations (>10 mg m^-3^) as a result of these species floating in the surface water [19,22].

Finally, the model developed in this study is currently restricted for the detection of *Ostreopsis sp*., *H. akashiwo*, *K. foliaceum*, *N. scintillans/miliaris*, *T. erythraeum* and *C. polykrikoides* in the Red Sea (see S1 File and S3 Fig for the spatial distribution of *C. polykrikoides*). Thus, future efforts could be directed towards strengthening training datasets and extending the model validation with more *in situ* datasets of other types of HABs that were not investigated in this study (for example, diatoms and other dinoflagellates species such as *P. bahamense* and *P. quinquecorne*). Furthermore, the inclusion of additional training datasets may allow the model to map the spatial extent of HAB species in other regions adjacent to the Red Sea, such as the Arabian Sea, Indian Ocean and Mediterranean Sea. In addition, we noticed some uncertainty in the accuracy of the present model when it detects blooms that are spatially less dense, and/or consist of mixed assemblages of different phytoplankton (especially cyanobacteria/diatoms with dinoflagellates). For example, the dinoflagellate *N. scintillans/miliaris*, which was observed at a low cell density (i.e., cell counts ≈ 4.4 ×10^3^ cells L^-1^; see Fig 10f—“st1”), often co-occurred with cyanobacteria/diatoms [14,19]. Hence, the optical properties of the water were influenced by the presence of both organisms [19,41]. Despite the aforementioned limitations and uncertainty, our results suggest that the proposed remote sensing model provide an adequate and efficient tool to detect the presence/absence of HABs, and monitor their spatial extents in the Red Sea.

## Conclusions

We presented a HAB detection model that combines satellite ocean color data and a second-order derivative technique to detect and map the spatial extent of different HAB events in the Red Sea. The performance of this model was evaluated with daily MODIS-Aqua images that were selected to coincide with HABs that have been previously reported in the basin. False color imagery and satellite-derived *Chl-a* maps were used to assess the spatial variation of these HAB species. Comparisons between modelled outputs and *in situ* field data further validated the ability of the remote sensing model to detect the presence/absence of HABs. At species concentrations greater than 1 mg m^-3^, the model was able to distinguish species-specific HABs in the coastal and open waters of the Red Sea. The accuracy of the model may vary depending on different spatio-temporal scales, as a result of the limited data used for training the second-order derivative technique. The model’s sensitivity is currently tailored towards the detection of ‘‘typical blooms”, on a specific day, corresponding to previously reported HAB events in the upper layer of the ocean. Thus, future efforts could be directed towards investigating the seasonality and interannual variability of HABs in the Red Sea over the last two decades. Such information is necessary for the implementation of aquaculture management strategies and the sustainability of regional economies in the coastal zone of the Red Sea.

## Supporting information

S1 Fig Examples of particulate absorption spectra derived from MODIS-Aqua data through existing bio-optical models for the six different HABs in the Red Sea waters. **(a)** *H*. *akashiwo* **(b)** *Ostreopsis sp*. **(c)** *K*. *foliaceum* **(d)** *N*. *scintillans/miliaris* **(e)** *C. polykrikoides* **(f)** *T*. *erythraeum*.
(TIF)

S2 Fig Examples of particulate backscattering spectra derived from MODIS-Aqua data through existing bio-optical models for the different HABs in the Red Sea waters. **a)** *H*. *akashiwo* **(b)** *Ostreopsis sp*. **(c)** *K*. *foliaceum* **(d)** *N*. *scintillans/miliaris* **(e)** *C. polykrikoides* **(f)** *T*. *erythraeum*.
(TIF)

S3 Fig Remotely sensing *C*. *polykrikoides* blooms in the southern Red Sea. **(a)** False color composite image generated using the NIR, green and blue reflectances (i.e., R = *R*_*rs*_ (748), G = *R*_*rs*_ (547), B = *R*_*rs*_ (443)). **(b)** Satellite derived *Chl-a* using ABI algorithm. **(c)** MODIS-Aqua-derived maps of the *C*. *polykrikoides* blooms in the Red Sea on 13 March 2010.
(TIF)

S1 File Accuracy assessment, particulate absorption and backscattering spectral analysis, and spatial distribution of *Cochlodinium polykrikoides* blooms in the Red Sea. (DOCX)
