# Supplementary figures and images for: Remotely sensing harmful algal blooms in the Red Sea
Source: PLoS One. 2019 Apr 16;14(4):e0215463. doi: 10.1371/journal.pone.0215463 (PMC6467414; doi:10.1371/journal.pone.0215463)

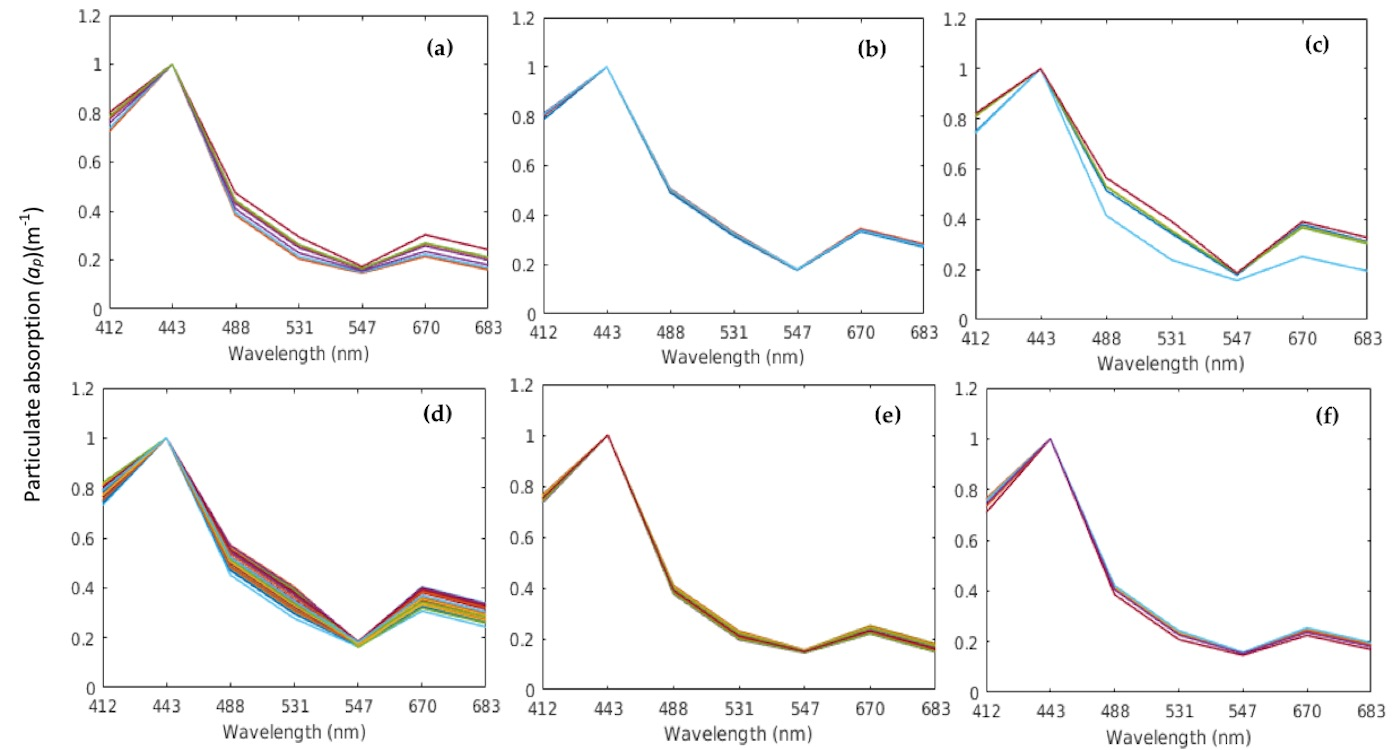

Supplement: S1 Fig — (a) H. akashiwo (b) Ostreopsis sp. (c) K. foliaceum (d) N. scintillans/miliaris (e) C. polykrikoides (f) T. erythraeum. (TIF) [file pone.0215463.s001.tif]

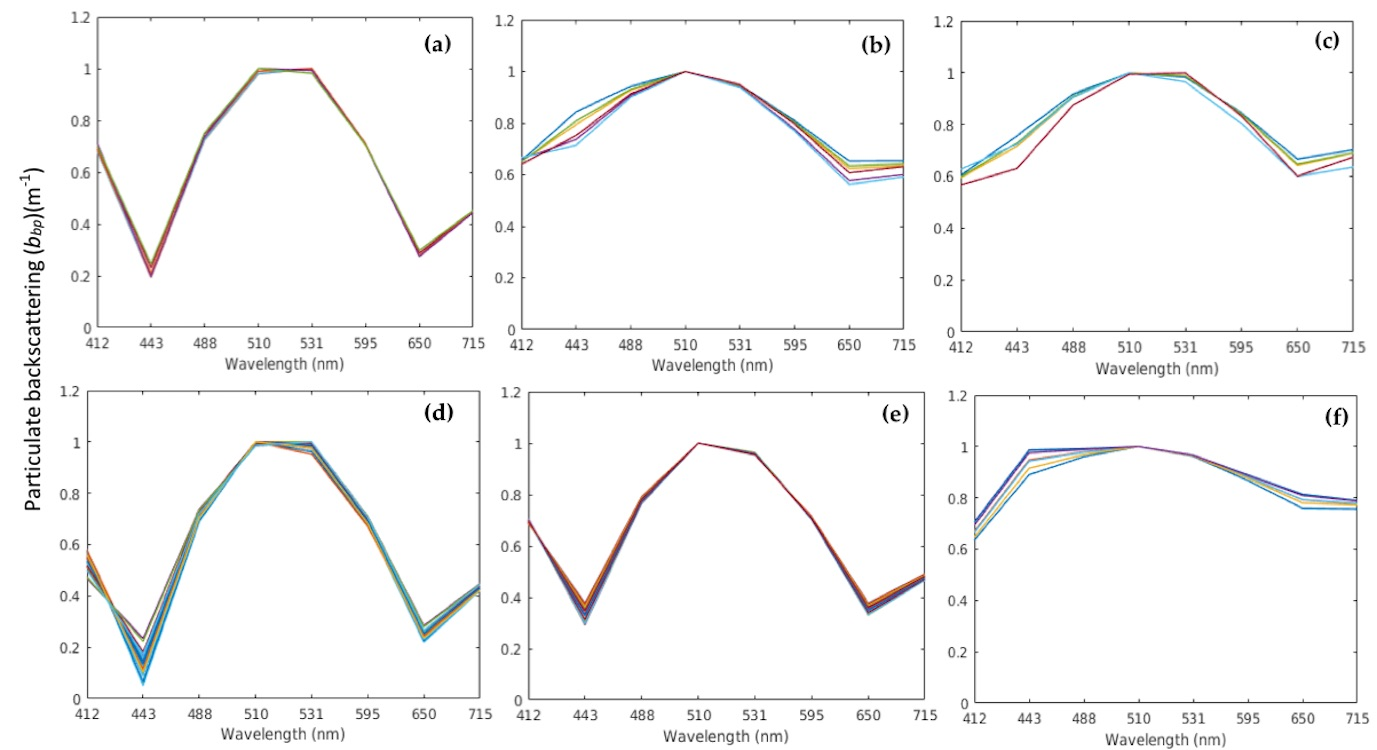

Supplement: S2 Fig — a) H. akashiwo (b) Ostreopsis sp. (c) K. foliaceum (d) N. scintillans/miliaris (e) C. polykrikoides (f) T. erythraeum. (TIF) [file pone.0215463.s002.tif]

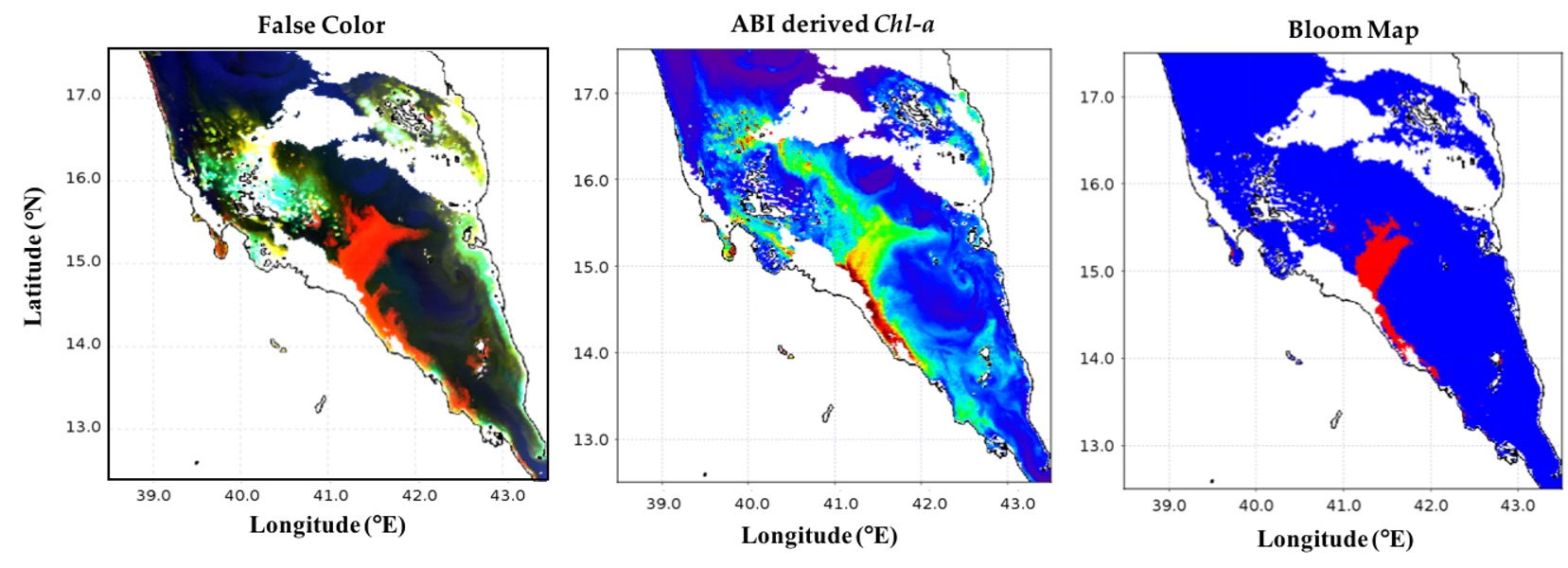

Supplement: S3 Fig — (a) False color composite image generated using the NIR, green and blue reflectances (i.e., R = Rrs (748), G = Rrs (547), B = Rrs (443)). (b) Satellite derived Chl-a using ABI algorithm. (c) MODIS-Aqua-derived maps of the C. polykrikoides blooms in the Red Sea on 13 March 2010. (TIF) [file pone.0215463.s003.tif]
