# Supplementary material for: Remotely sensing harmful algal blooms in the Red Sea
Source: PLoS One. 2019 Apr 16;14(4):e0215463. doi: 10.1371/journal.pone.0215463 (PMC6467414; doi:10.1371/journal.pone.0215463)
Supplement: S1 File — (DOCX) [file pone.0215463.s004.docx]

**Accuracy Assessment**

The accuracy of HAB detection and classifications is generally assessed by taking bloom pixel from the satellite imagery and checking their accuracy against reference data. A measure for the overall classification accuracy can be estimated by counting how many pixels were classified the same in the satellite image and in the reference data (i.e., the sum of the major diagonal being correctly classified) and dividing this by the total number of pixels:

$Overall accuracy = \left( \frac{D}{N} \right)\times100\%$ (1)

D: Total number of correct classifications,

N: Total number of classifications.

The main downfall of this measure is that it does not assess the accuracy of individual classifications. To overcome this limitation, user and producer accuracy were widely used to assess the accuracy the individual classifications [1]. The user accuracy is computed using the number of correctly classified pixels divided by total number of pixels assigned to a particular category.

$User's accuracy = \left( \frac{D}{R} \right)\times100\%$ (2)

R: Number in row total.

The User’s Accuracy was computed for each row;

The producer’s accuracy informs the image analyst of the number of pixels correctly classified in a particular category as a percentage of the total number of pixels actually belonging to that category in the image.

$Producer's accuracy = \left( \frac{D}{C} \right)\times100\%$ (3)

C: Number in column total.

The Producer’s Accuracy was computed for each column. The map of accuracy can also be estimated from Kappa coefficient. It is a measure of agreement between model predictions and reference data. This measure uses all elements in the matrix and not just the diagonal ones.

Kappa Coefficient, $\hat{K}=\left( \frac{NA-B}{N^{2}-B} \right)\times100\%$ (4)

A = the sum of r diagonal elements,

B = sum of the r products (row total × column total).

N = number of pixels in the error matrix

**Particulate absorption and backscattering spectral analysis**

The particulate absorption spectra of all the different species have two sharp peaks ~ 443 nm and ~ 670 nm. The magnitude of these peaks varies depending on the Chl-a concentration of the HAB species [2]. The Chl-a absorption peaks at ~ 443 nm and ~ 670 nm for these species, as well as the enhanced backscattering features in the green and NIR, can clearly be observed in S1-S2 Figs. Hu et al. [3] revealed that particulate absorption spectra of *T. erythraeum* display peaks at 443 and 670 nm, but often exhibit other minor absorption features at 495, 545, and 565 nm. The latter two peaks correspond to phycoerythrobilin (PEB) and the first peak corresponds to phycourobilin (PUB). Since MODIS-Aqua derived spectra average the absorption coefficient of phytoplankton within each pixel, they typically represent two primary peaks at 443 and 670 nm (see S1 Fig).

**Spatial distribution of Cochlodinium polykrikoides blooms in the Red Sea**

We processed the MODIS-Aqua data acquired on cloud free day of 13 March 2010 for generating the results of C. polykrikoides blooms in southern Red Sea waters. S3 Fig showed an aggravated bloom of C. polykrikoides covering a wide area in the coastal waters of southern Red Sea. In the false color composite MODIS image, the dark red features show evidence of the presence of C. polykrikoides blooms in the southern Red Sea waters characterized by much stronger radiances in the red and NIR wavelength bands. We observed that the ABI measurements indicated patches of high chlorophyll (>2 mg m^-3^). Furthermore, ABI algorithm along with the present remote sensing model captured apparent patterns of C. polykrikoides bloom and clearly distinguishes from other naturally occurring features in the southern Red sea waters.

**References**

1. Simon A, Shanmugam P. An algorithm for classification of algal blooms using MODIS-Aqua data in oceanic waters around India. Advances in Remote Sensing. 2012; 1(2).
2. Gokul EA, Shanmugam P. An optical system for detecting and describing major algal blooms in coastal and oceanic waters around India. Journal of Geophysical Research: Oceans. 2016;121(6): 4097-4127.
3. Hu C, Cannizzaro J, Carder KL, Muller-Karger FE, Hardy R. Remote detection of *Trichodesmium* blooms in optically complex coastal waters: Examples with MODIS full-spectral data. Remote Sensing of Environment. 2010; 15, 114(9): 2048-2058.
